# Validation of a Novel Noninvasive Technology to Estimate Blood Oxygen Saturation Using Green Light: Observational Study

**DOI:** 10.2196/46974

**Published:** 2024-03-27

**Authors:** Sanjay Gokhale, Vinoop Daggubati, Georgios Alexandrakis

**Affiliations:** 1 Department of Biomedical Engineering The University of Texas at Arlington Arlington, TX United States; 2 Shani Biotechnologies LLC Austin, TX United States

**Keywords:** reflectance spectroscopy, tissue oxygen measurements, oxygen saturation, pulse oximeter, oxyhemoglobin concentration, oxygen level, racial disparity

## Abstract

**Background:**

Pulse oximeters work within the red-infrared wavelengths. Therefore, these oximeters produce erratic results in dark-skinned subjects and in subjects with cold extremities. Pulse oximetry is routinely performed in patients with fever; however, an elevation in body temperature decreases the affinity of hemoglobin for oxygen, causing a drop in oxygen saturation or oxyhemoglobin concentrations.

**Objective:**

We aimed to determine whether our new investigational device, the Shani device or SH1 (US Patent 11191460), detects a drop in oxygen saturation or a decrease in oxyhemoglobin concentrations.

**Methods:**

An observational study (phase 1) was performed in two separate groups to validate measurements of hemoglobin and oxygen concentrations, including 39 participants recruited among current university students and staff aged 20-40 years. All volunteers completed baseline readings using the SH1 device and the commercially available Food and Drug Administration–approved pulse oximeter Masimo. SH1 uses two light-emitting diodes in which the emitted wavelengths match with absorption peaks of oxyhemoglobin (hemoglobin combined with oxygen) and deoxyhemoglobin (hemoglobin without oxygen or reduced hemoglobin). Total hemoglobin was calculated as the sum of oxyhemoglobin and deoxyhemoglobin. Subsequently, 16 subjects completed the “heat jacket study” and the others completed the “blood donation study.” Masimo was consistently used on the finger for comparison. The melanin level was accounted for using the von Luschan skin color scale (VLS) and a specifically designed algorithm. We here focus on the results of the heat jacket study, in which the subject wore a double-layered heated jacket and pair of trousers including a network of polythene tubules along with an inlet and outlet. Warm water was circulated to increase the body temperature by 0.5-0.8 °C above the baseline body temperature. We expected a slight drop in oxyhemoglobin concentrations in the heating phase at the tissue level.

**Results:**

The mean age of the participants was 24.1 (SD 0.8) years. The skin tone varied from 12 to 36 on the VLS, representing a uniform distribution with one-third of the participants having fair skin, brown skin, and dark skin, respectively. Using a specific algorithm and software, the reflection ratio for oxyhemoglobin was displayed on the screen of the device along with direct hemoglobin values. The SH1 device picked up more minor changes in oxyhemoglobin levels after a change in body temperature compared to the pulse oximeter, with a maximum drop in oxyhemoglobin concentration detected of 6.5% and 2.54%, respectively.

**Conclusions:**

Our new investigational device SH1 measures oxygen saturation at the tissue level by reflectance spectroscopy using green wavelengths. This device fared well regardless of skin color. This device can thus eliminate racial disparity in these key biomarker assessments. Moreover, since the light is shone on the wrist, SH1 can be readily miniaturized into a wearable device.

## Introduction

Pulse oximetry is routinely performed in all patients with elevated body temperature. However, high blood temperature decreases the affinity of oxygen for hemoglobin (Hb) [1], and an elevation in temperature by approximately 1 °C decreases arterial oxygen saturation (sO_2_) by only 0.5% [2]. Therefore, the decrease is very minimal and is not clinically significant in subjects with a baseline sO_2_ level within the normal range [3].

Blood sO_2_ measured by pulse oximetry is currently used to monitor tissue hypoxia. This method uses red and infrared wavelengths in the light spectrum and there is no correction for the level of skin melanin. Consequently, the estimated values are often inaccurate in people with darker skin tones due to the overlapping absorption spectra of melanin [4,5]. Administrative and health authorities have also recognized this issue; however, a solution has not yet been put forward [6].

To overcome this limitation, we have developed a novel technology to estimate Hb and tissue oxygenation. The scientific basis, details, and underlying technology of the device are published elsewhere [7]. In brief, the Shani device (SH1) measures reflectance of light from the skin by a pair of light-emitting diodes (LEDs), displayed as the reflectance ratio from LED1 (E1) and LED2 (E2) and as the sum of the ratios (E; E1+E2). The method of measurement is described in our previous report [7]. The light is shone on the wrist and only the reflected light is picked up by the sensors. This analog signal is then converted to a digital format by the processor, which can be analyzed, displayed, and stored in digital form (US Patent 11191460). In this device and associated technology, the reflectance ratio varies inversely with the concentration of Hb or Hb combined with oxygen (OxyHb) [8].

With an increase in body temperature, a slight drop in OxyHb is expected. Here, we focus on the results of the phase 1 study (heat jacket study) to validate the device in healthy human volunteers. The body temperature of the participants was increased by circulating warm water in a double-layered heat jacket; therefore, a slight drop in the OxyHb concentration at the tissue level was expected during the heating phase. The aim of this study was to determine whether our new device can detect the drop in sO_2_ or a decrease in tissue OxyHb concentrations.

## Methods

### Investigational Device

**Figure 1:**
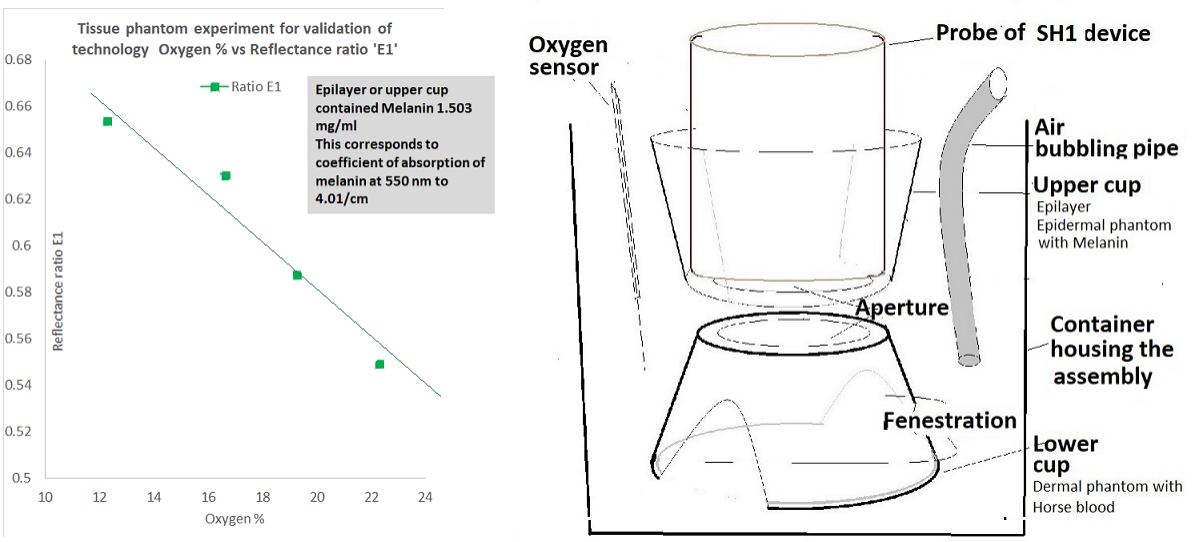
Results (left) and setup (right) of the tissue phantom experiment [9]. The graph shows the relationship between the reflectance ratio of light-emitting diode 1 (E1) with the measured oxygen saturation level (%). The epilayer or upper cup contained 1.503 mg/ml melanin, corresponding to a coefficient of melanin absorption (at 550 nm) of 4.01/cm. This figure was adapted from Gokhale et al [9] which is published under Creative Commons Attribution 4.0 International License [10].

The SH1 device was validated in tissue phantom experiments. Here, we are presenting the results of the phase 1 heat jacket study in healthy human volunteers. Details of preclinical studies and the results of these experiments have been recently published elsewhere [9]. In the preclinical experiments, we used synthetic melanin as an epilayer mimicking melanin and we used horse blood in the lower layer corresponding to the dermis of the skin. As a part of preclinical studies, we performed an absorption scan of synthetic melanin (Sigma-Aldrich, Instrument-Infinite 200 and BME089922 software system). At a melanin concentration of 1.5 mg/ml, the absorption coefficient of melanin (ie, the absorption of light per unit length) was determined to be 4.01/ml. Even with this high melanin concentration in the epilayer, our device could detect changes in OxyHb levels in the lower dermal layer. Dark-skinned subjects showed an absorption coefficient of melanin at 550 nm, corresponding to 2.5/ml. Therefore, in this preclinical study, we tested melanin levels that are higher (darker) than those measured for dark-skinned subjects. The relationship of melanin concentration with the absorption coefficient of melanin at a wavelength of 550 nm was determined in the previous study [9]. This new investigational device (SH1) measures Hb by shining light from LED1 and LED2 sequentially to obtain measures of both OxyHb and reduced Hb (ie, DeoxyHb). This is termed the “hemoglobin mode” of the operation. The reflectance ratio for LED1 is referred to as E1 and that for LED2 is referred to as E2. The reflectance ratio E1 corresponds to OxyHb, which is inversely related to the oxygen content (Figure 1). The device could detect changes in oxygen concentration in the blood, even in the presence of high melanin in the epilayer, thereby mimicking the detection of hypoxia in dark-skinned subjects.

### Eligibility and Recruitment

As per the institutional policy, only current students and members of the staff of University of Texas at Arlington were recruited for this validation study. External candidates, including past students, were not allowed to participate. The age restriction for participation was 20-40 years. For recruitment, flyers were sent by email and posters were displayed in designated locations, including the lobby, near elevators, the cafeteria, and common rooms.

The phase 1 study was performed with two separate cohorts independently for validation of measurements of Hb and oxygen concentrations. There were 39 participants in the two studies; there was only one staff member and the remaining participants were students. Both studies included baseline measurements at visit 1. All 39 volunteers completed baseline readings using our new investigational SH1 device. Subsequently, 16 participants completed the heat jacket study and the others were included in the blood donation study. The commercially available pulse oximeter Masimo was used on the finger for comparison. Skin melanin was accounted for using the von Luschan skin color scale (VLS) and a specifically designed algorithm. Skin tone measurements were performed by two observers independently and the mean value was noted and rounded to the nearest integer. We here focus only on the results of the heat jacket study.

### Ethical Considerations

The heat jacket study was approved by the institutional review board (IRB) at The University of Texas at Arlington (STU-2021-0150; approval date February 23, 2021). Written informed consent was obtained from each participant. Copies of consent forms are maintained by IRB authorities. The privacy and confidentiality of the participants were respected and data are stored in a coded, anonymous format. Each participant received monetary compensation as per the stipulated rules and regulations laid out by the IRB of The University of Texas at Arlington.

### Study Design

The heat jacket study included 16 volunteers and was performed under the IRB-approved protocol. At visit 1, baseline measurements of Hb and oxygen concentrations were taken using our novel SH1 device and the commercial pulse oximeter Masimo. At visit 2, after obtaining appropriate consent, the baseline demographic information was obtained. The participant was then asked to wear a double-layered heat jacket and a pair of trousers, which comprise a network of polythene tubules and an inlet and outlet. Subsequently, the participant swallowed a telemetry pill with some water; this is a small pill-shaped electronic object that is used to sense temperature. After a few minutes, the pill reaches the stomach, measures internal body temperature, and emits a signal. A sensor attached to the jacket receives these signals, which are then relayed to a monitor via a cable. Baseline readings were taken with the participant lying down. Warm water was circulated in the jacket and trousers to increase the body temperature by 0.5-0.8 °C above the baseline body temperature as measured with the telemetry pill. This increase in body temperature simulates clinically relevant fever settings. The heating phase lasted for 40 minutes, followed by cooling for the next 20 minutes. Cooling was achieved by circulating cold water through the jacket. Baseline and serial readings were taken with the new investigational SH1 device at 10-minute intervals. The measurements taken with the Masimo Pronto pulse oximeter were used for comparison. A total of 7 sets of observations were obtained for each participant over a period of 60 minutes. Readings in the heating phase and cooling phase were rescaled for each participant with baseline measurements taken as 100%. The percentage drop in sO_2_ or the difference between maximum and minimum readings by the SH1 and Masimo devices was plotted for each participant. 

## Results

**Figure 2:**
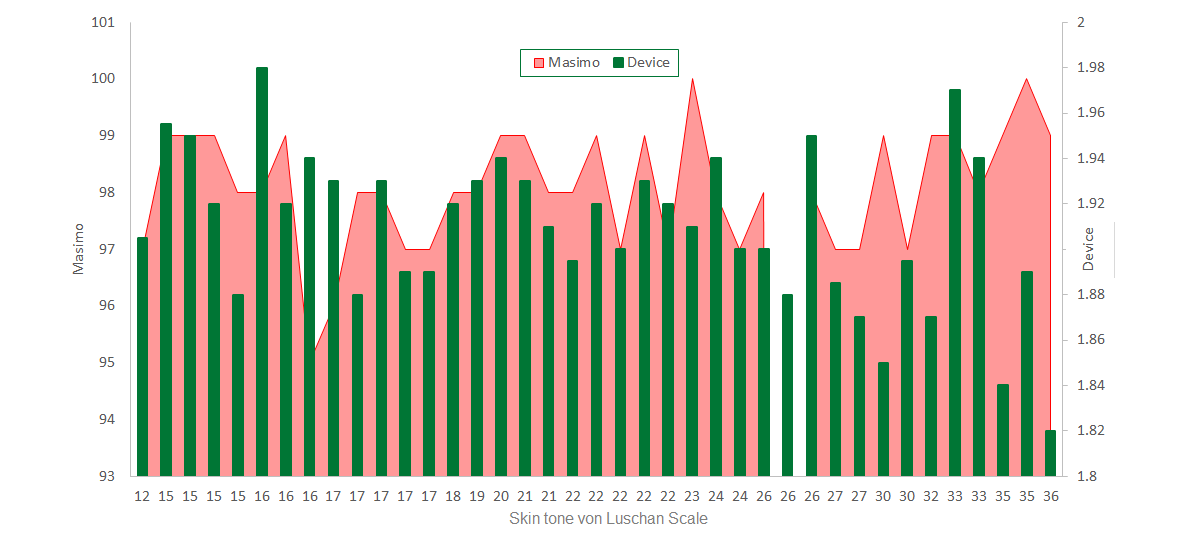
Skin tone versus oxygen saturation measured by the Shani device and Masimo pulse oximeter at baseline in all 39 participants. VLS: von Luschan skin coloration scale.

The mean age of the study population was 24.1 (SD 0.8) years. The skin tone varied from 12 to 36 on the VLS with a uniform distribution: one-third of the participants had fair skin, brown skin, and dark skin, respectively. Using a specific algorithm accounting for melanin, as determined from the VLS, and associated software, the reflection ratio for OxyHb is displayed on the screen along with direct Hb values [11]. We had baseline readings for all 39 participants (Figure 2) with our SH1 device and the commercial pulse oximeter Masimo. As seen in Figure 2, baseline oxygen concentrations of all 39 participants as measured by SH1 device readings fell within a range similar to those measured by the pulse oximeter.

**Figure 3:**
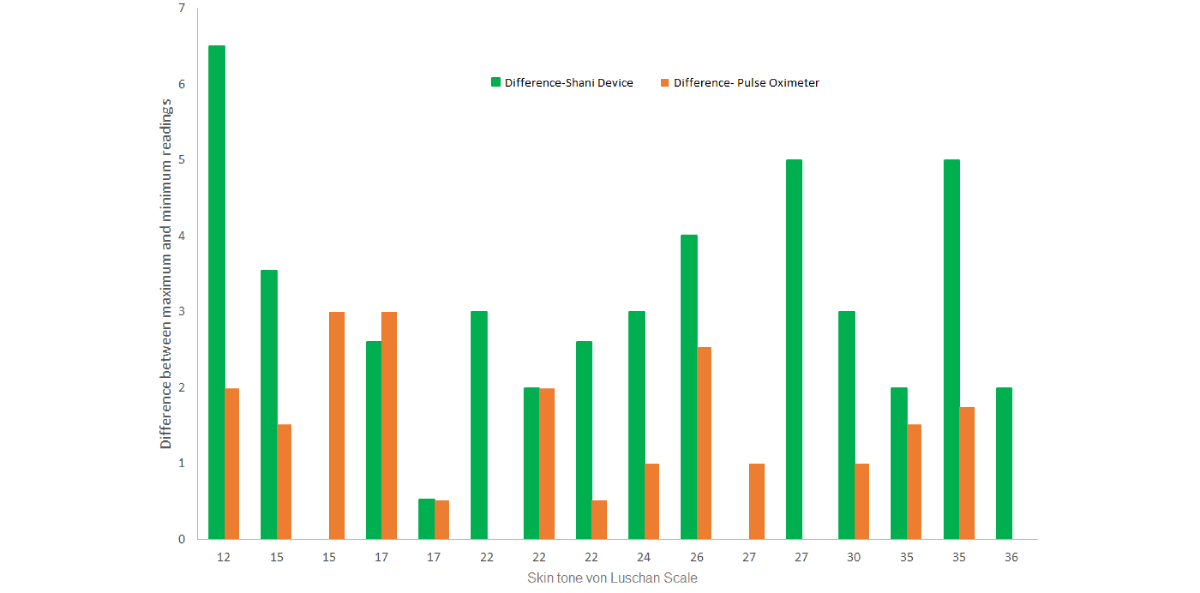
Skin tone versus the percentage drop in oxygen concentrations for the 16 participants in the heat jacket study, as measured by the new device and the commercial pulse oximeter Masimo. VLS: von Luschan skin color scale.

In the heat jacket study, we expected a slight drop in OxyHb concentrations in the heating phase at the tissue level. Our hypothesis was confirmed after analyzing the data for the reflection ratios E1, E2, and E (E1+E2), followed by computation of OxyHb and total Hb values. Figure 3 shows the distribution of the skin tone (according to the VLS) of the participants compared to the percentage drop in oxygen concentrations in the 16 subjects participating in the heat jacket study. Our device could pick up more minor changes in OxyHb levels after a change in body temperature than possible with the pulse oximeter. The maximum drop in OxyHb concentrations picked up by our device was 6.5% compared to a drop of only 2.54% sensed by the pulse oximeter. The average change in OxyHb measured by our device was 2.98%, whereas that of the pulse oximeter was 1.33%, with a median of 3% and 1%, respectively.

## Discussion

### Principal Findings

The baseline readings taken by the SH1 device (Figure 2) while the participants were lying in supine position showed some interesting features, including different readings for subjects with the same skin tone (eg, subjects with skin tones 15, 16, 22, and 26 on the VLS) and similar readings for subjects with different skin tones (eg, subjects with skin tones 15, 16, and 26 on the VLS). Therefore, the baseline measurement of tissue oxygen levels (ie, OxyHb levels) as determined by the SH1 device is independent of skin tone and likely depends on tissue metabolism and the basal metabolic rate.

In this heat jacket study, all participants showed a drop in OxyHb levels during the heating phase, followed by a rise in the cooling phase. In some cases, the rise was slightly higher than baseline levels.

We compared the difference in maximum and minimum readings obtained by our SH1 device and the commercial pulse oximeter Masimo. Figure 3 shows the distribution of the difference across different skin tones. This drop in oxygen saturation was magnified by the SH1 device compared to the readings obtained with the pulse oximeter. The difference between maximum and minimum readings by the SH1 device was 6.5%, whereas that for Masimo was only 2.54%.

As stated earlier, the decrease in arterial oxygen levels with fever is very small, even in patients in the intensive care unit, and this drop in sO_2_ is not clinically significant in patients with baseline sO_2_ within the normal range. Nonetheless, this minor change is picked up well by our device and is not detected by the pulse oximeter. The commercial pulse oximeter Masimo Pronto measures arterial sO_2_ by photoplethysmography and red-infrared wavelengths [12], whereas the novel SH1 device measures sO_2_ at the tissue level by reflectance spectroscopy and using two green wavelengths. These are two important fundamental differences between these technologies. Table 1 compares the properties of the SH1 device and pulse oximeter.

**Table 1:** Comparison of the properties of the new investigational device (Shani device) and a conventional pulse oximeter.

Properties	Pulse oximeter	Shani device
Radiation used	Red and infrared wavelengths	Green visible light
Method	Transmittance of light	Reflectance of light
Any solution for melanin interference?	None so far	Yes; accounted for using a special algorithm
Accuracy of O_2_% in dark-skinned subjects?	Doubtful; erratic results are obtained with darker skin tones	High accuracy in all subjects, regardless of skin color
Diagnostic ability (eg, hypotension, shock)	Poor accuracy	High accuracy
Continuous monitoring possible	Yes	Yes
Site of testing	Fingertip	Back of wrist
Can be transitioned into a wearable?	Unclear	Certainly
Data storage and transfer	Yes	Yes
Battery operated	Yes	Yes

### Working of the Device

**Figure 4:**
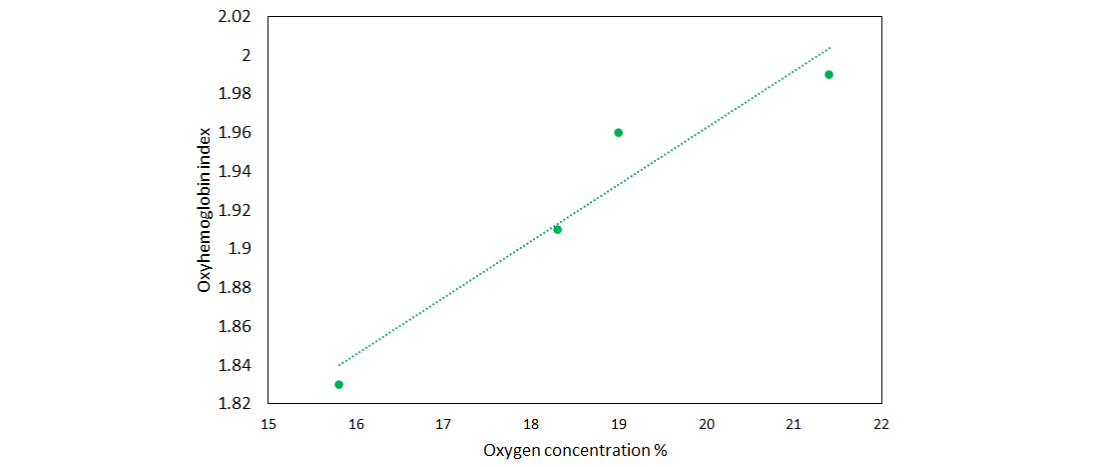
Oxygen concentrations (%) versus the oxyhemoglobin index.

The emitted wavelengths of the two LEDs matched with the absorption peaks of OxyHb and DeoxyHb (ie, reduced Hb) between 520 nm and 580 nm. As mentioned above, in our new device, the input from LED1 and LED2 of the probe is received as an analog signal. This signal is then converted to digital format by the processor, which can be analyzed, displayed, and stored in digital form. In tissue phantom experiments, we bubbled air and measured the increase in oxygen concentrations in horse blood using an oxygen sensor [9]. The reflection ratio E1 received from LED1 correlates inversely with OxyHb, E2 from LED2 correlates with DeoxyHb, and E (sum of E1 and E2) correlates with total Hb. After analyzing data from the tissue phantom experiments, we derived another new parameter termed the “OxyHb index” as a measure of OxyHb concentration. In our earlier tissue phantom experiments [9], we found that this OxyHb index varies directly with the oxygen concentration in the blood (Figure 4). This means that low oxygen concentrations are reflected as a low OxyHb index and vice versa.

Tissue oxygenation parameters include the concentrations of Hb and OxyHb in the tissue [13]. Tissue sO_2_ monitoring is a relatively new technology, and a drop in tissue sO_2_ is an early warning sign of peripheral hypoperfusion and the onset of tissue hypoxia [14]. All oximeters currently available using red and infrared wavelengths to target arterial blood flow, which measures sO_2_ in the conducting vascular/arterial system. Our device targets the capillary-venous network and measures tissue oxygenation. Sepsis and shock result in disturbances in microcirculatory perfusion and a change in tissue oxygen utilization that may not be reflected in arterial sO_2_ levels. According to many authorities, tissue oxygenation is a better marker of the underlying pathological processes as well as responsiveness to some treatments [15,16]. Tissue oxygen levels are more important, because the arterial O_2_ content and an adequate bulk transport of oxygen by the cardiovascular system may not guarantee delivery of oxygen to the critical tissues of the body [17]. Additionally, tissue Hb sO_2_ has been determined to be a better predictor of the multiorgan failure outcome [18]. Near-infrared spectroscopy also has well-known limitations [19]. To overcome these limitations, our new device uses green light (520-570 nm) for the estimation of Hb and OxyHb, while accounting for the impact of skin color on the measurements. A summary presentation displays relevant information about this technology in Multimedia Appendices 1 and 2. The video demonstrates the working of this new device.

Using green wavelengths for measurements of Hb and oxygen concentrations with a special algorithm to account for melanin is a novel concept and our efforts have already been appreciated by experts in the field [20,21]. In this study, we have used the VLS for the measurements of skin tone. This is an interval scale with measurements such as 18, 23, and 36. However, we are developing a technology to quantify melanin in the skin more precisely in a noninvasive manner, providing measurements on a continuous scale (eg, 15.25, 17.50, and 30.75). This method is patented and under development.

### Strengths and Limitations

The main limitation of this study is the small sample size. Although our device fared better than the pulse oximeter, larger studies are needed in patients with diverse skin tones and a variable degree of tissue hypoxia for further clinical development of the device.

The main strength of this study is that the SH1 device offers an early warning system. A drop in tissue sO_2_ is an early warning sign of peripheral hypoperfusion and the onset of tissue hypoxia. Our device can sense this before traditional oximeters can raise the alarm, thereby demonstrating the potential for saving lives.

Our device can also be used in diverse settings, from home monitoring to intensive care, making critical data readily available. Light is shone on the wrist of the individual and is then measured. Therefore, in the future, this device can be miniaturized for wearable technology. The device can be operated in either hemoglobin or oximetry mode. In hemoglobin mode, the device can be used for noninvasive measurements of Hb, whereas in oximetry mode, the device can be used for the continuous monitoring of sO_2_.

Racial equity is another important advantage of our device. Unlike most pulse oximeters on the market, our device works for all skin tones, eliminating bias and improving care for people of color.

The specific technology underlying the design of our device offers specific advantages. First, the use of green light (520-570 nm) can obtain information from the microcirculation, revealing tissue oxygen levels invisible to red and infrared wavelengths of light. Second, our unique algorithm accounts for variations in melanin, ensuring accurate readings. These advantages can consequently lead to improved outcomes, as early detection and tailored therapies lead to better health for all, especially marginalized groups. Moreover, the ability to obtain a faster diagnosis and intervention can save both resources and lives. Finally, the device offers global reach as it is affordable and adaptable, thereby demonstrating potential to improve health care in resource-limited settings.

### Conclusion

The changes in sO_2_ at the tissue level in normoxemic subjects are very minor or minimal. Current pulse oximeters, limited to red and infrared wavelengths, only capture the “big picture” of arterial blood flow by measuring oxygen saturation in the conducting arterial system while missing critical changes in the microcirculation, where sepsis and shock wreak havoc, before the arterial oxygen dips.

Our device is a game-changer for measuring tissue oxygenation by shining green light and offering a more sensitive marker of these hidden dangers. Our device was validated to accurately measure tissue oxygen levels and could pick up very minor changes after a change in body temperature in the heat jacket study, demonstrating improved performance compared to the commercial pulse oximeter. Tissue oxygenation parameters include the concentrations of Hb and OxyHb in the tissue. Our device worked better and appeared to be more sensitive than the pulse oximeter even for subjects with light skin or skin tone (eg, VLS 12-18). Since the sample size of this study was small, additional studies with large sample size, a diverse population, and varied degree of hypoxia are required for further confirmation.

## Appendix

Multimedia Appendix 1 Summary Presentation

Multimedia Appendix 2 Shani Biotech Demo(10mb)

## Abbreviations

DeoxyHb: deoxyhemoglobin
Hb: hemoglobin
IRB: institutional review board
LED: light-emitting diode
OxyHb: oxyhemoglobin
SH1: Shani device
sO2: oxygen saturation
VLS: von Luschan skin color scale

## Data Availability

Since this is a patented technology, the data cannot be widely shared. However, the data are available with the institutional review board of the University of Texas at Arlington and can be accessed through Shani Biotechnologies LLC after completing nondisclosure agreement or confidential disclosure agreement formalities.
