# Supplementary material for: Validation of a Novel Noninvasive Technology to Estimate Blood Oxygen Saturation Using Green Light: Observational Study
Source: JMIR Biomed Eng. 2024 Mar 27;9:e46974. doi: 10.2196/46974 (PMC11041477; doi:10.2196/46974)
Supplement: Multimedia Appendix 1 [file biomedeng_v9i1e46974_app1.pptx]

## Slide 1
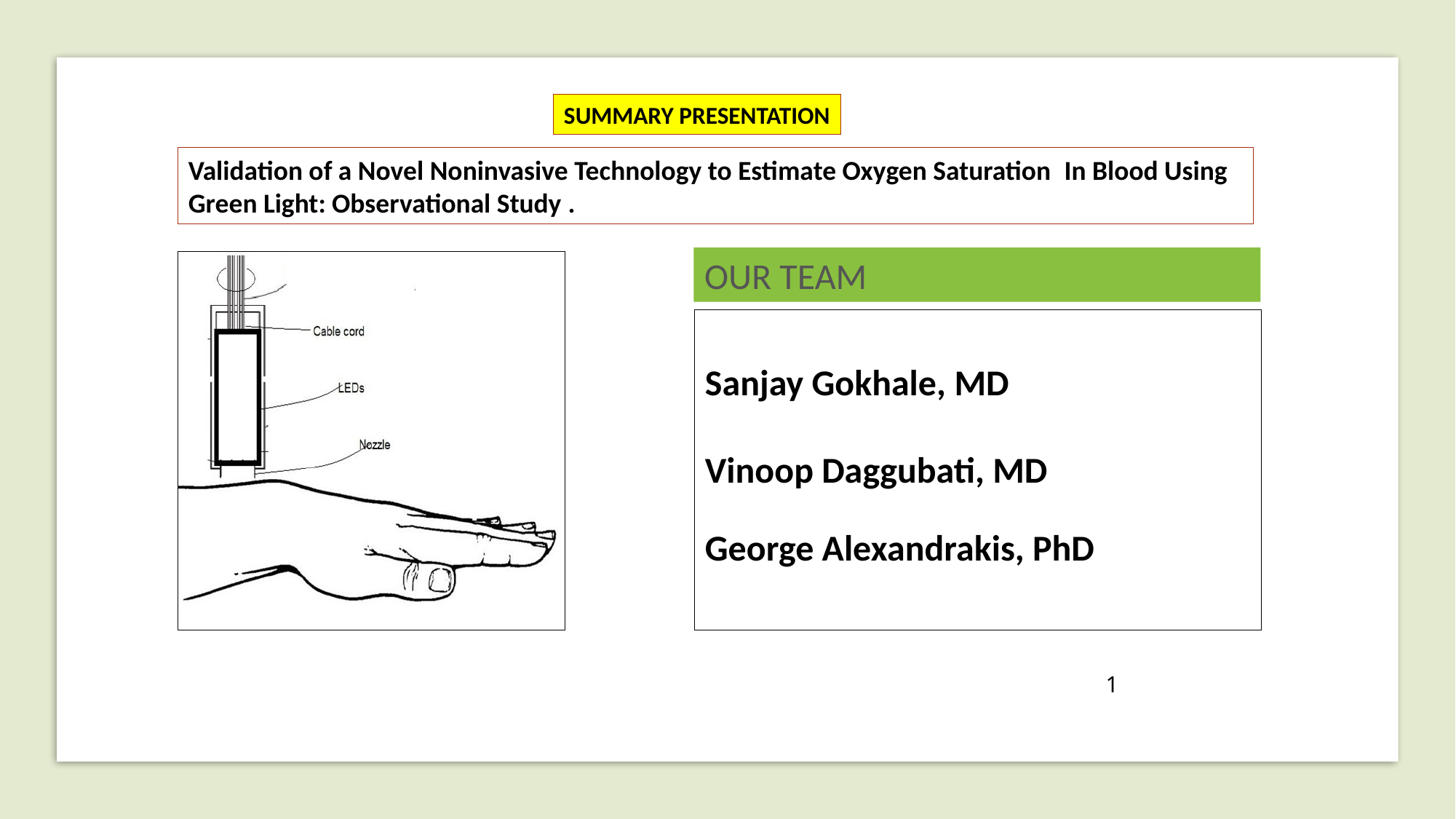

SUMMARY PRESENTATION
Validation of a Novel Noninvasive Technology to Estimate Oxygen Saturation  In Blood Using Green Light: Observational Study .
OUR TEAM
Sanjay Gokhale, MD
Vinoop Daggubati, MD
George Alexandrakis, PhD
1
1

## Slide 2
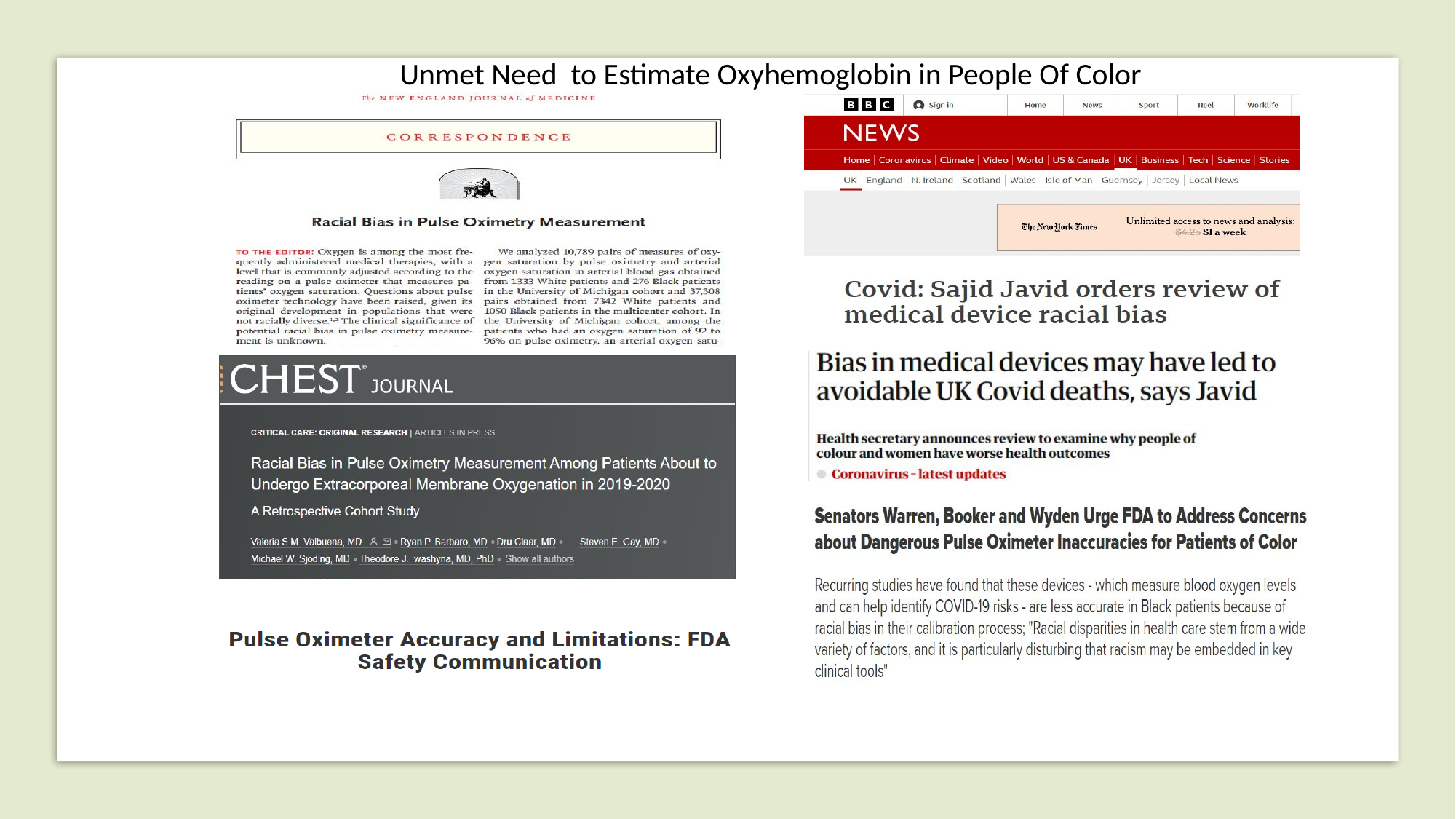

Unmet Need to Estimate Oxyhemoglobin in People Of Color
2

## Slide 3
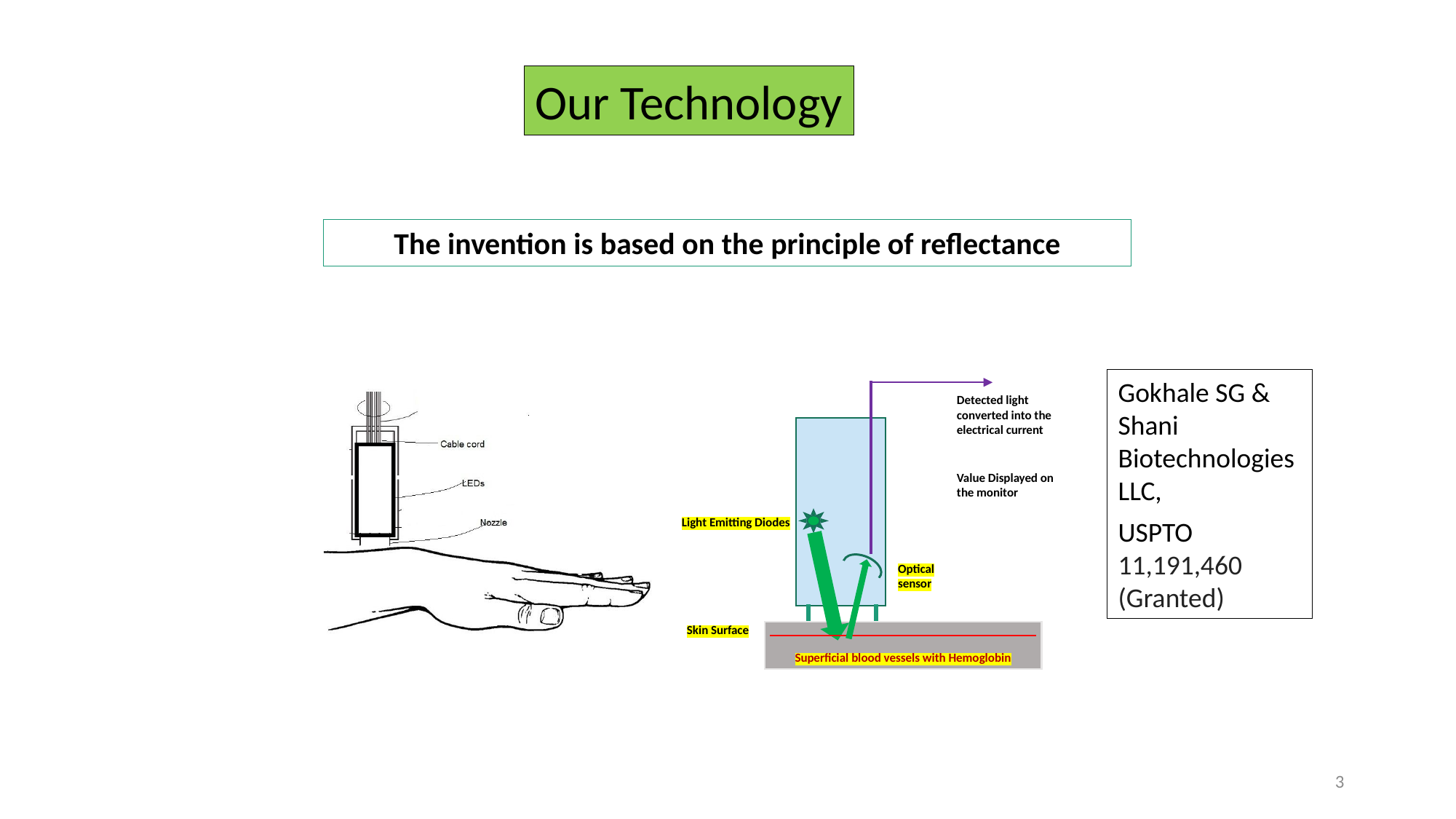

Our Technology
# The invention is based on the principle of reflectance
Gokhale SG & Shani Biotechnologies LLC,
USPTO 11,191,460 (Granted)
Detected light converted into the electrical current
Value Displayed on the monitor
Light Emitting Diodes
Superficial blood vessels with Hemoglobin
Optical sensor
Skin Surface
3

## Slide 4
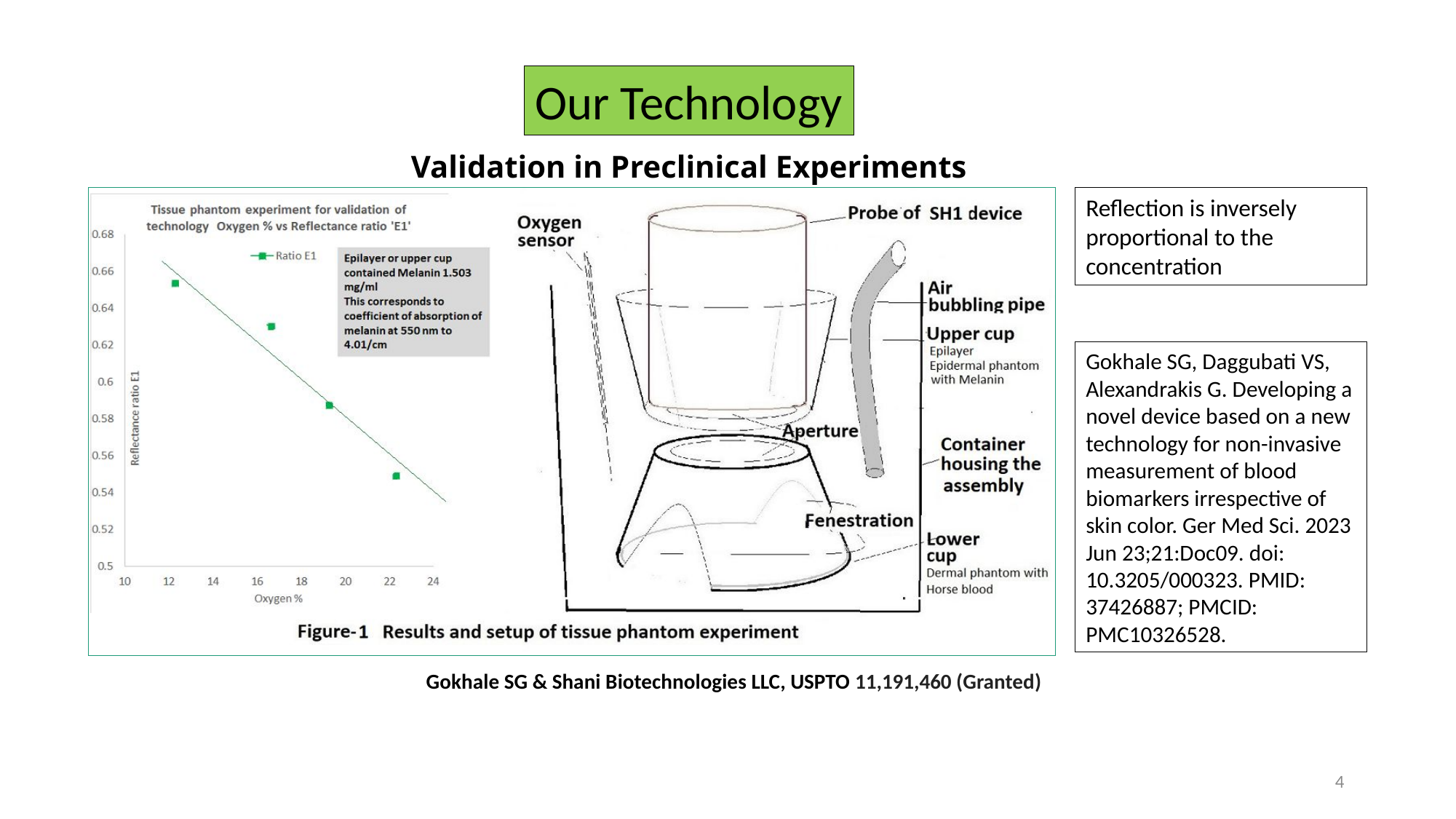

Our Technology
# Validation in Preclinical Experiments
Reflection is inversely proportional to the concentration
Gokhale SG, Daggubati VS, Alexandrakis G. Developing a novel device based on a new technology for non-invasive measurement of blood biomarkers irrespective of skin color. Ger Med Sci. 2023 Jun 23;21:Doc09. doi: 10.3205/000323. PMID: 37426887; PMCID: PMC10326528.
Gokhale SG & Shani Biotechnologies LLC, USPTO 11,191,460 (Granted)
4

## Slide 5
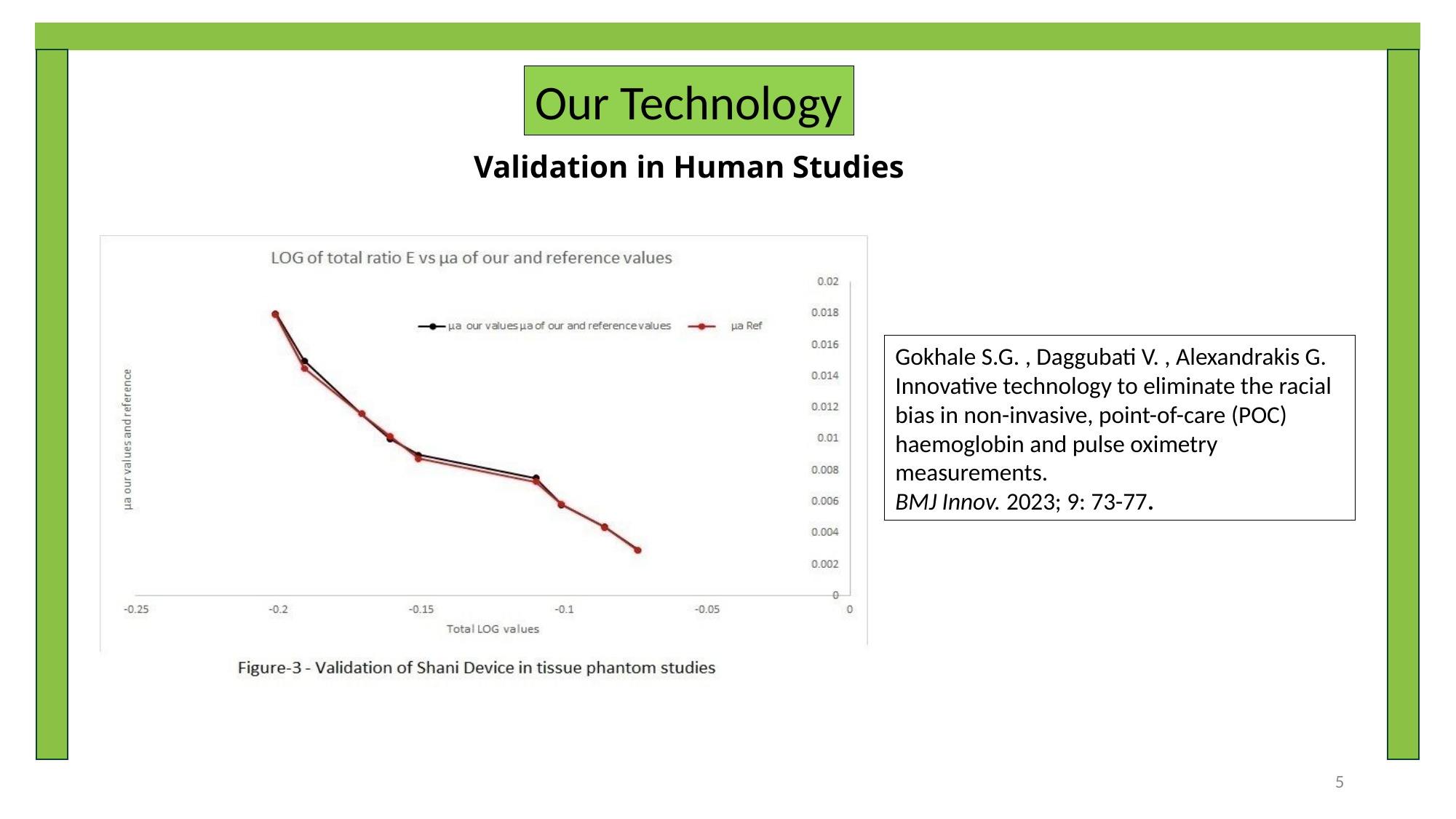

Our Technology
# Validation in Human Studies
Gokhale S.G. , Daggubati V. , Alexandrakis G.
Innovative technology to eliminate the racial bias in non-invasive, point-of-care (POC) haemoglobin and pulse oximetry measurements.
BMJ Innov. 2023; 9: 73-77.
5

## Slide 6
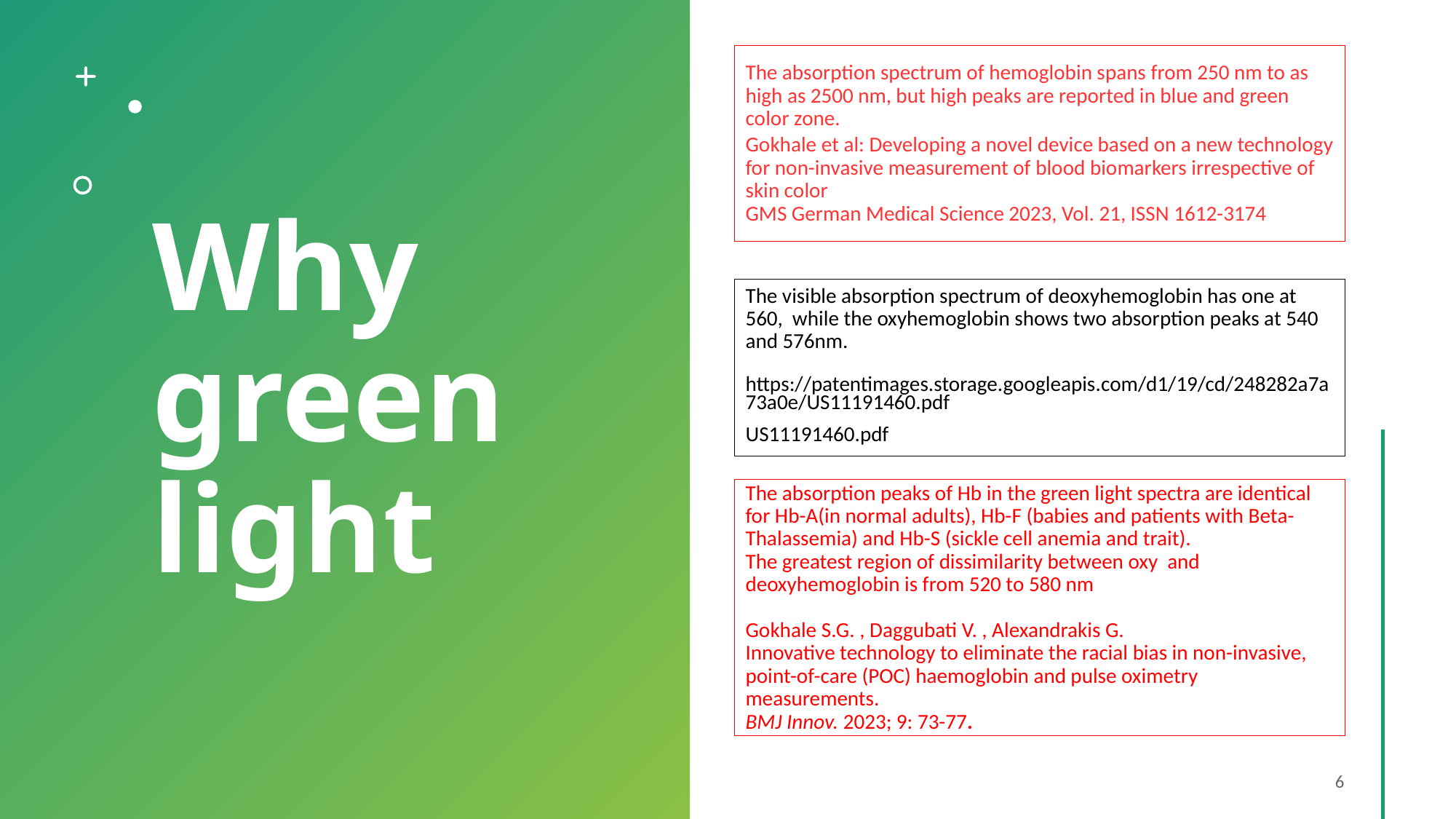

Why green light
The absorption spectrum of hemoglobin spans from 250 nm to as high as 2500 nm, but high peaks are reported in blue and green color zone.
Gokhale et al: Developing a novel device based on a new technology for non-invasive measurement of blood biomarkers irrespective of skin color
GMS German Medical Science 2023, Vol. 21, ISSN 1612-3174
The visible absorption spectrum of deoxyhemoglobin has one at 560, while the oxyhemoglobin shows two absorption peaks at 540 and 576nm.
https://patentimages.storage.googleapis.com/d1/19/cd/248282a7a73a0e/US11191460.pdf
US11191460.pdf
The absorption peaks of Hb in the green light spectra are identical for Hb-A(in normal adults), Hb-F (babies and patients with Beta-Thalassemia) and Hb-S (sickle cell anemia and trait).
The greatest region of dissimilarity between oxy and deoxyhemoglobin is from 520 to 580 nm
Gokhale S.G. , Daggubati V. , Alexandrakis G.
Innovative technology to eliminate the racial bias in non-invasive, point-of-care (POC) haemoglobin and pulse oximetry measurements.
BMJ Innov. 2023; 9: 73-77.
6

## Slide 7
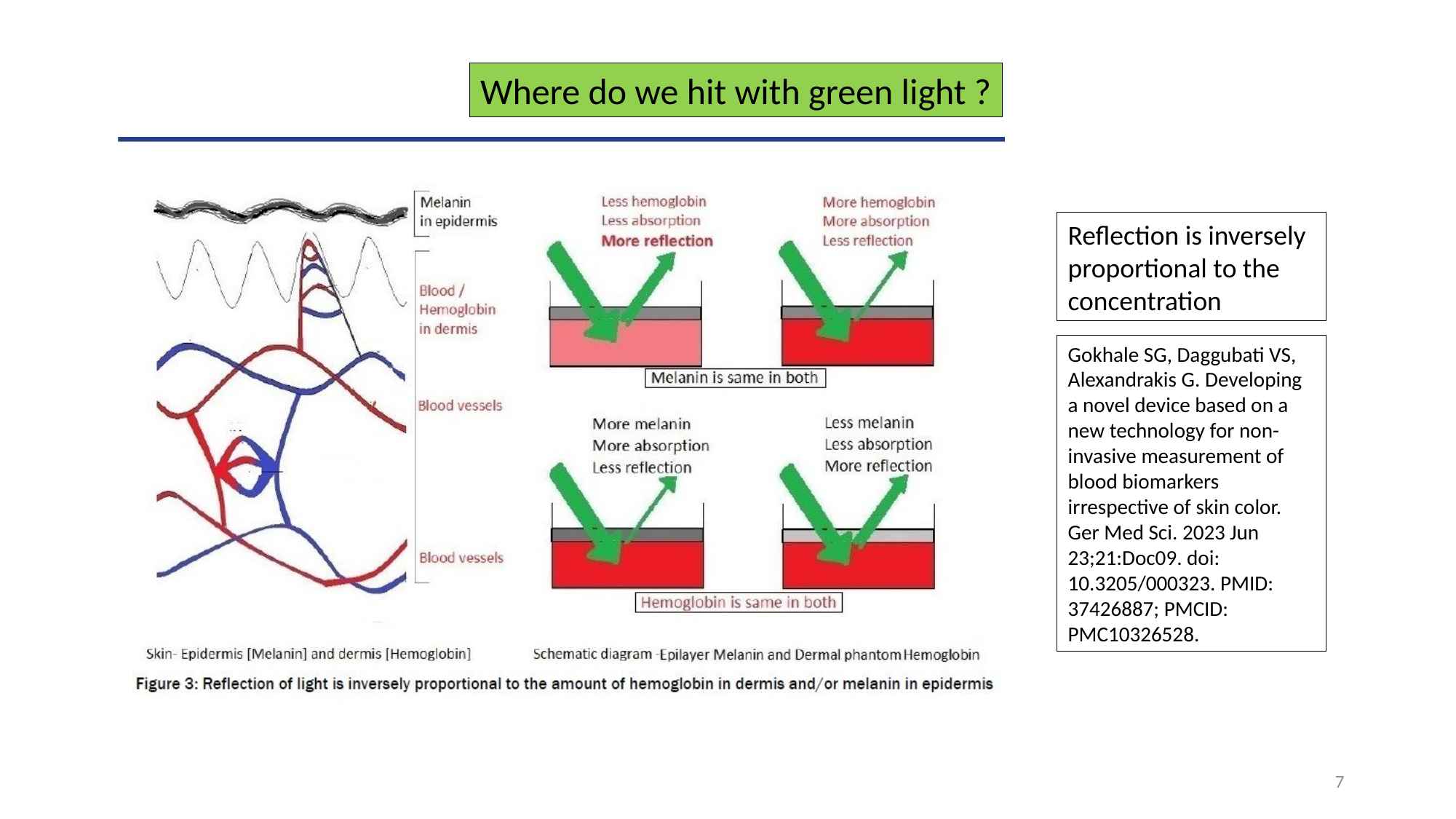

Where do we hit with green light ?
Reflection is inversely proportional to the concentration
Gokhale SG, Daggubati VS, Alexandrakis G. Developing a novel device based on a new technology for non-invasive measurement of blood biomarkers irrespective of skin color. Ger Med Sci. 2023 Jun 23;21:Doc09. doi: 10.3205/000323. PMID: 37426887; PMCID: PMC10326528.
7

## Slide 8
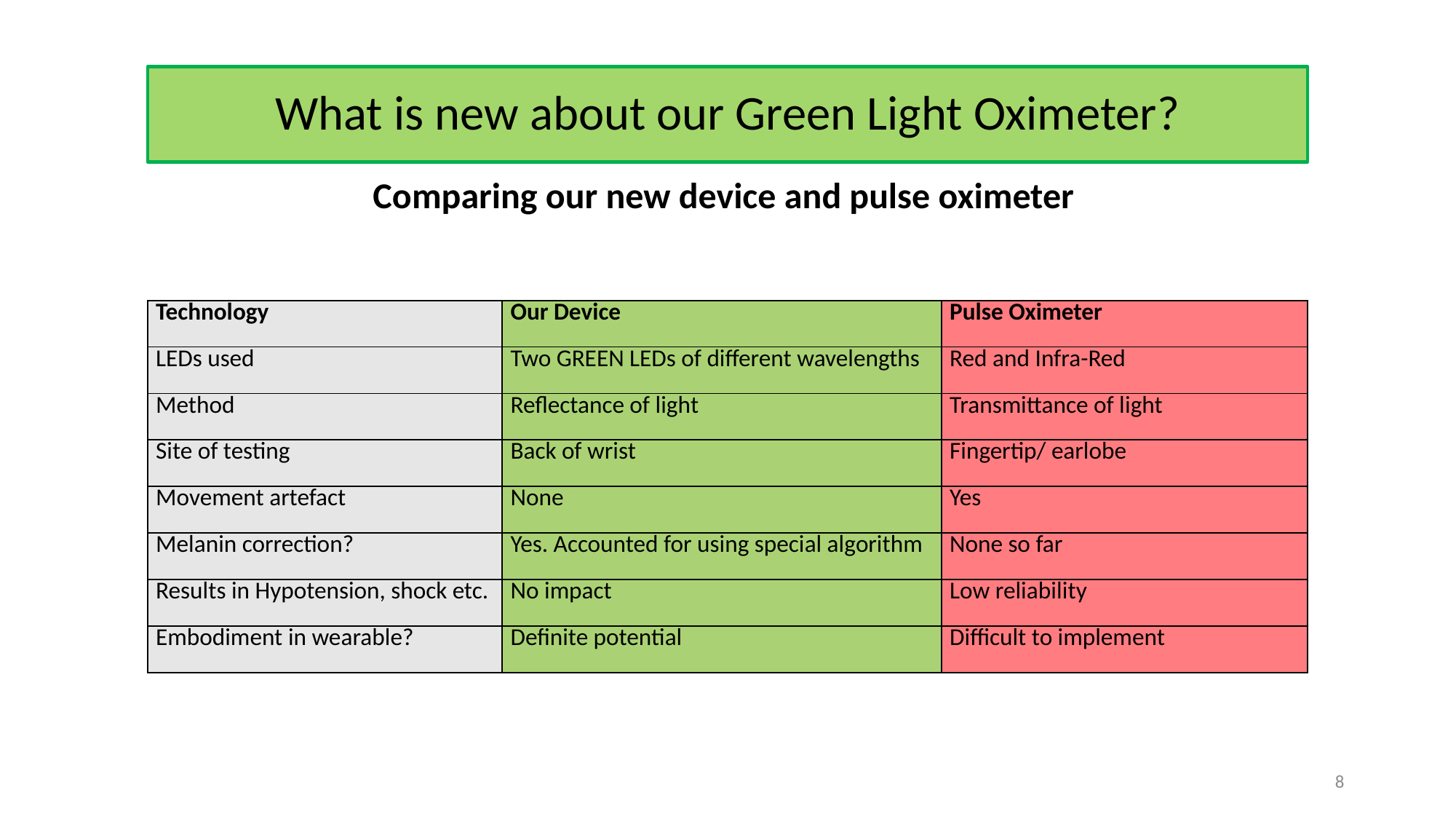

What is new about our Green Light Oximeter?
Comparing our new device and pulse oximeter
| Technology | Our Device | Pulse Oximeter |
| --- | --- | --- |
| LEDs used | Two GREEN LEDs of different wavelengths | Red and Infra-Red |
| Method | Reflectance of light | Transmittance of light |
| Site of testing | Back of wrist | Fingertip/ earlobe |
| Movement artefact | None | Yes |
| Melanin correction? | Yes. Accounted for using special algorithm | None so far |
| Results in Hypotension, shock etc. | No impact | Low reliability |
| Embodiment in wearable? | Definite potential | Difficult to implement |
8

## Slide 9
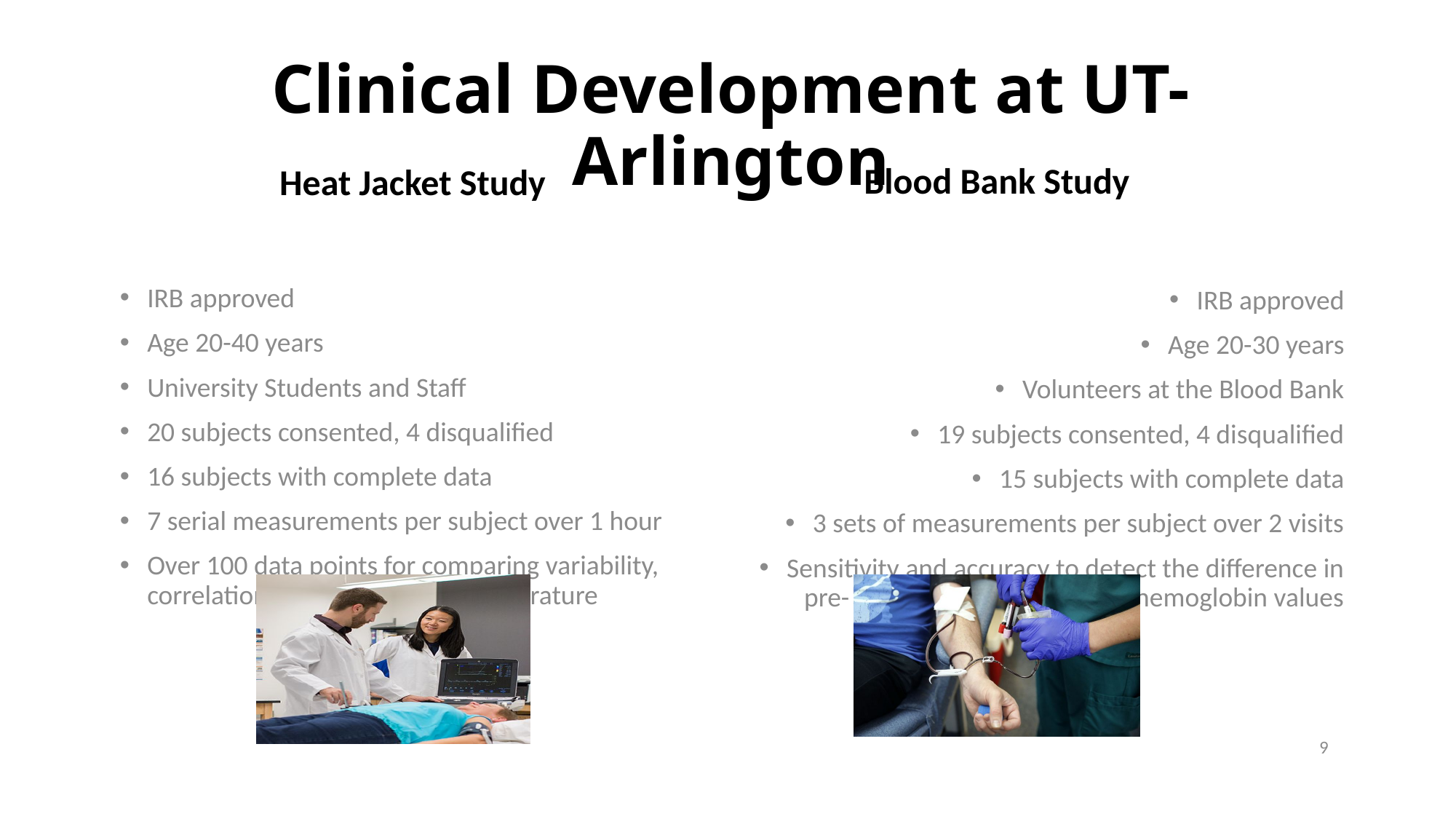

# Clinical Development at UT-Arlington
Heat Jacket Study
Blood Bank Study
IRB approved
Age 20-40 years
University Students and Staff
20 subjects consented, 4 disqualified
16 subjects with complete data
7 serial measurements per subject over 1 hour
Over 100 data points for comparing variability, correlation & effect of body temperature
IRB approved
Age 20-30 years
Volunteers at the Blood Bank
19 subjects consented, 4 disqualified
15 subjects with complete data
3 sets of measurements per subject over 2 visits
Sensitivity and accuracy to detect the difference in pre- and post-blood donation hemoglobin values
9

## Slide 10
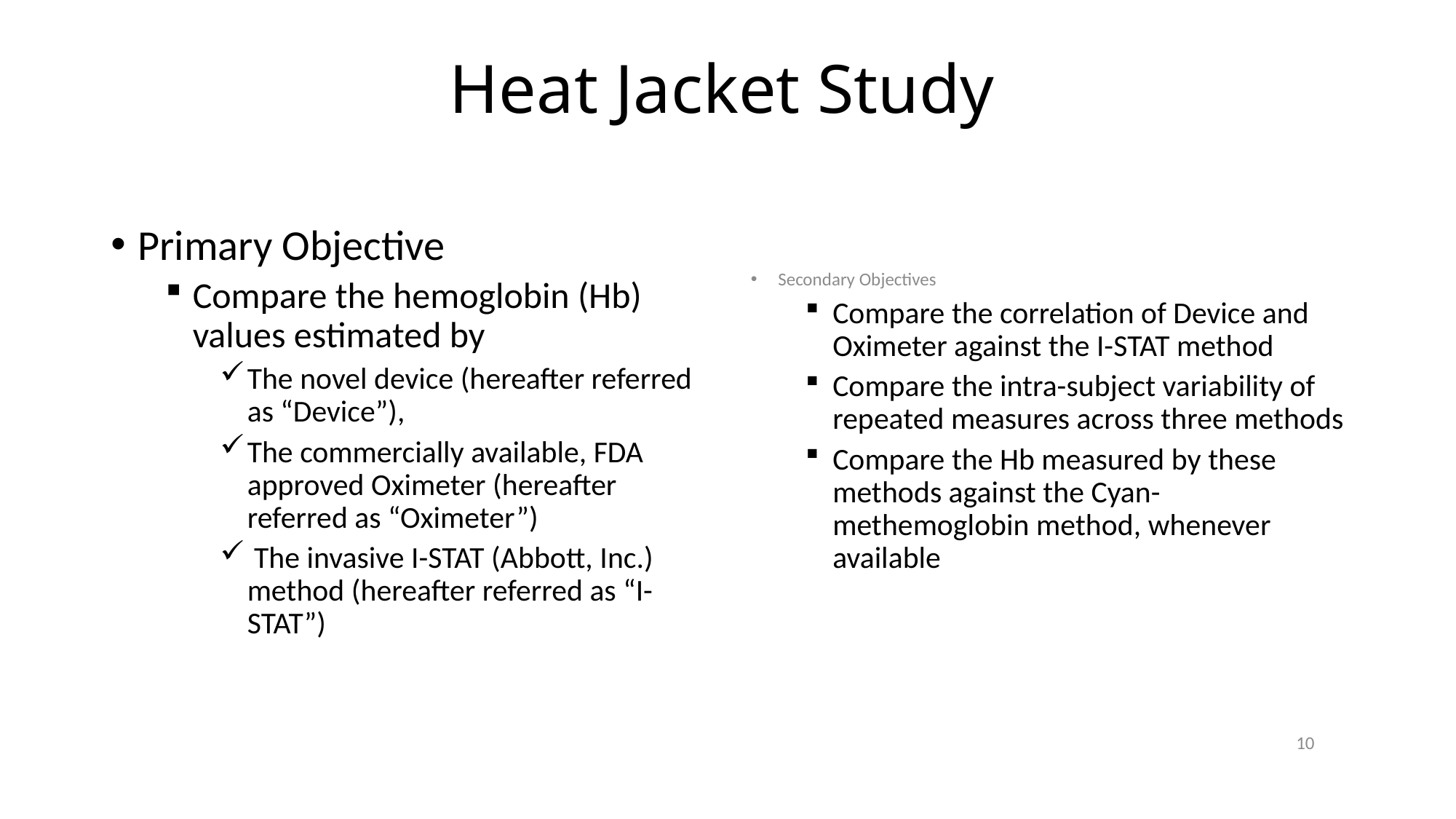

Heat Jacket Study
Primary Objective
Compare the hemoglobin (Hb) values estimated by
The novel device (hereafter referred as “Device”),
The commercially available, FDA approved Oximeter (hereafter referred as “Oximeter”)
 The invasive I-STAT (Abbott, Inc.) method (hereafter referred as “I-STAT”)
Secondary Objectives
Compare the correlation of Device and Oximeter against the I-STAT method
Compare the intra-subject variability of repeated measures across three methods
Compare the Hb measured by these methods against the Cyan-methemoglobin method, whenever available
10

## Slide 11
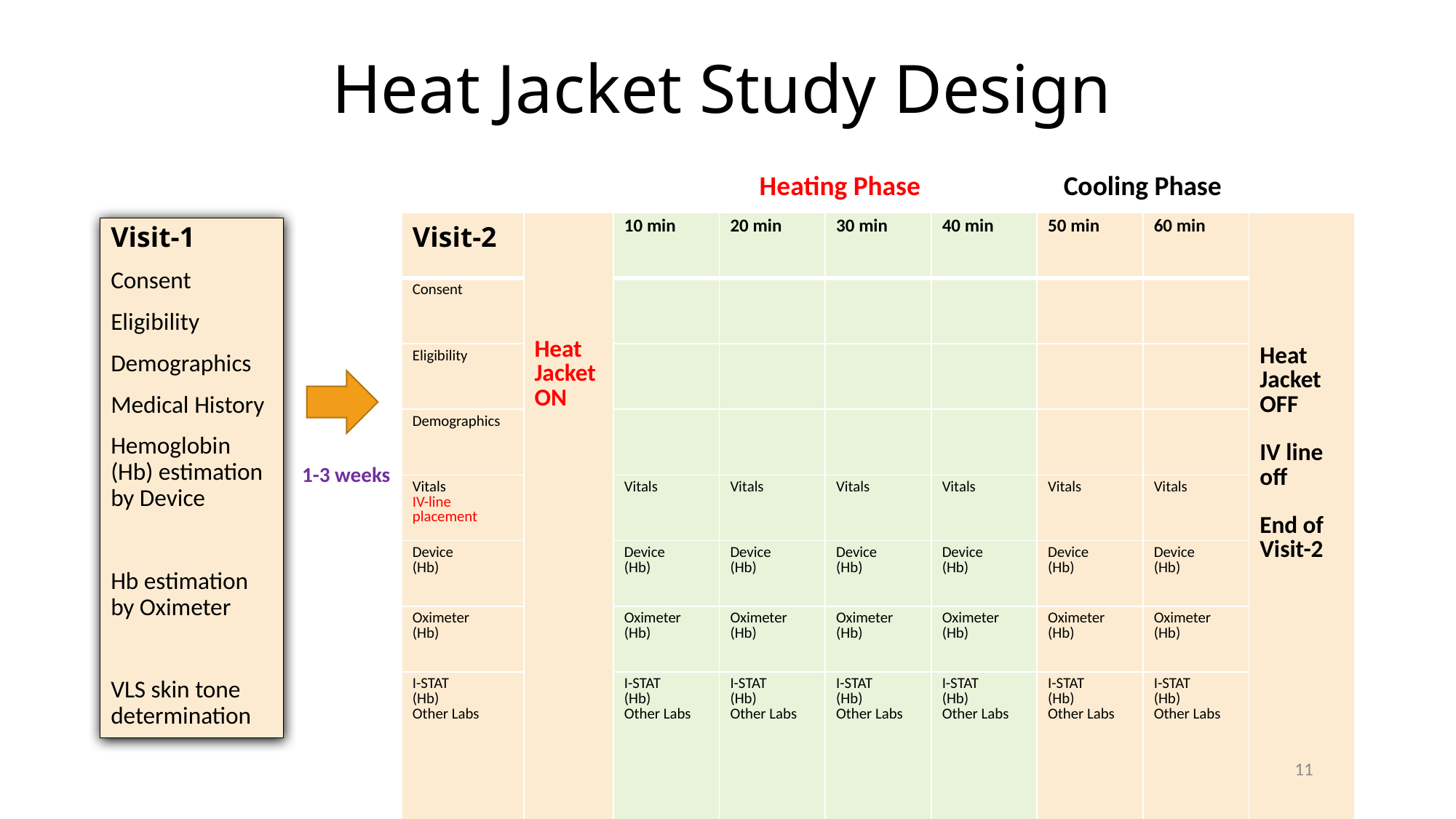

Heat Jacket Study Design
Heating Phase
Cooling Phase
| Visit-2 | Heat Jacket ON | 10 min | 20 min | 30 min | 40 min | 50 min | 60 min | Heat Jacket OFF IV line off End of Visit-2 |
| --- | --- | --- | --- | --- | --- | --- | --- | --- |
| Consent | | | | | | | | |
| Eligibility | | | | | | | | |
| Demographics | | | | | | | | |
| Vitals IV-line placement | Heat Jacket ON | Vitals | Vitals | Vitals | Vitals | Vitals | Vitals | |
| Device (Hb) | | Device (Hb) | Device (Hb) | Device (Hb) | Device (Hb) | Device (Hb) | Device (Hb) | |
| Oximeter (Hb) | | Oximeter (Hb) | Oximeter (Hb) | Oximeter (Hb) | Oximeter (Hb) | Oximeter (Hb) | Oximeter (Hb) | |
| I-STAT (Hb) Other Labs | | I-STAT (Hb) Other Labs | I-STAT (Hb) Other Labs | I-STAT (Hb) Other Labs | I-STAT (Hb) Other Labs | I-STAT (Hb) Other Labs | I-STAT (Hb) Other Labs | |
Visit-1
Consent
Eligibility
Demographics
Medical History
Hemoglobin (Hb) estimation by Device
Hb estimation by Oximeter
VLS skin tone determination
1-3 weeks
11

## Slide 12
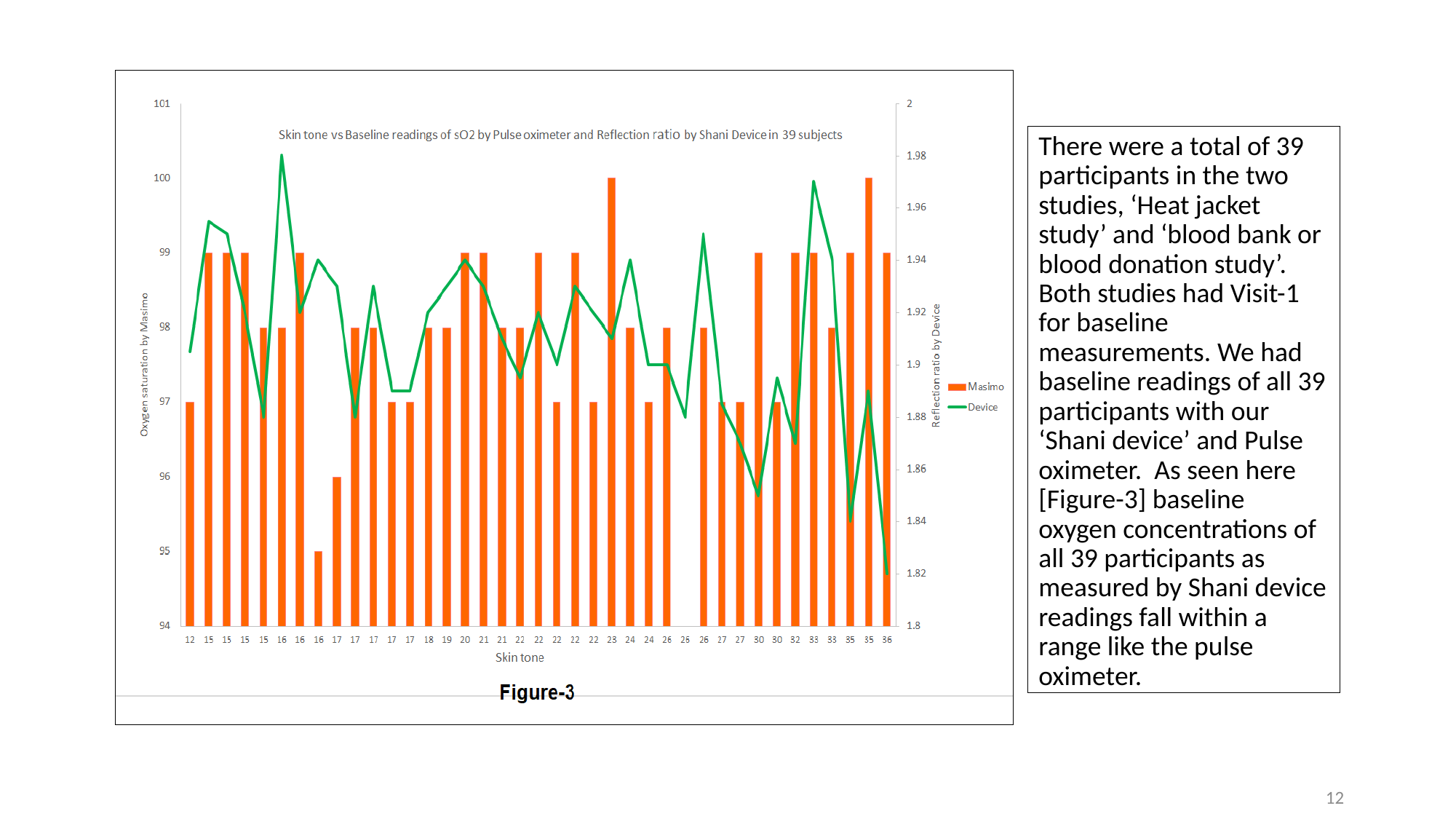

There were a total of 39 participants in the two studies, ‘Heat jacket study’ and ‘blood bank or blood donation study’. Both studies had Visit-1 for baseline measurements. We had baseline readings of all 39 participants with our ‘Shani device’ and Pulse oximeter. As seen here [Figure-3] baseline oxygen concentrations of all 39 participants as measured by Shani device readings fall within a range like the pulse oximeter.
12

## Slide 13
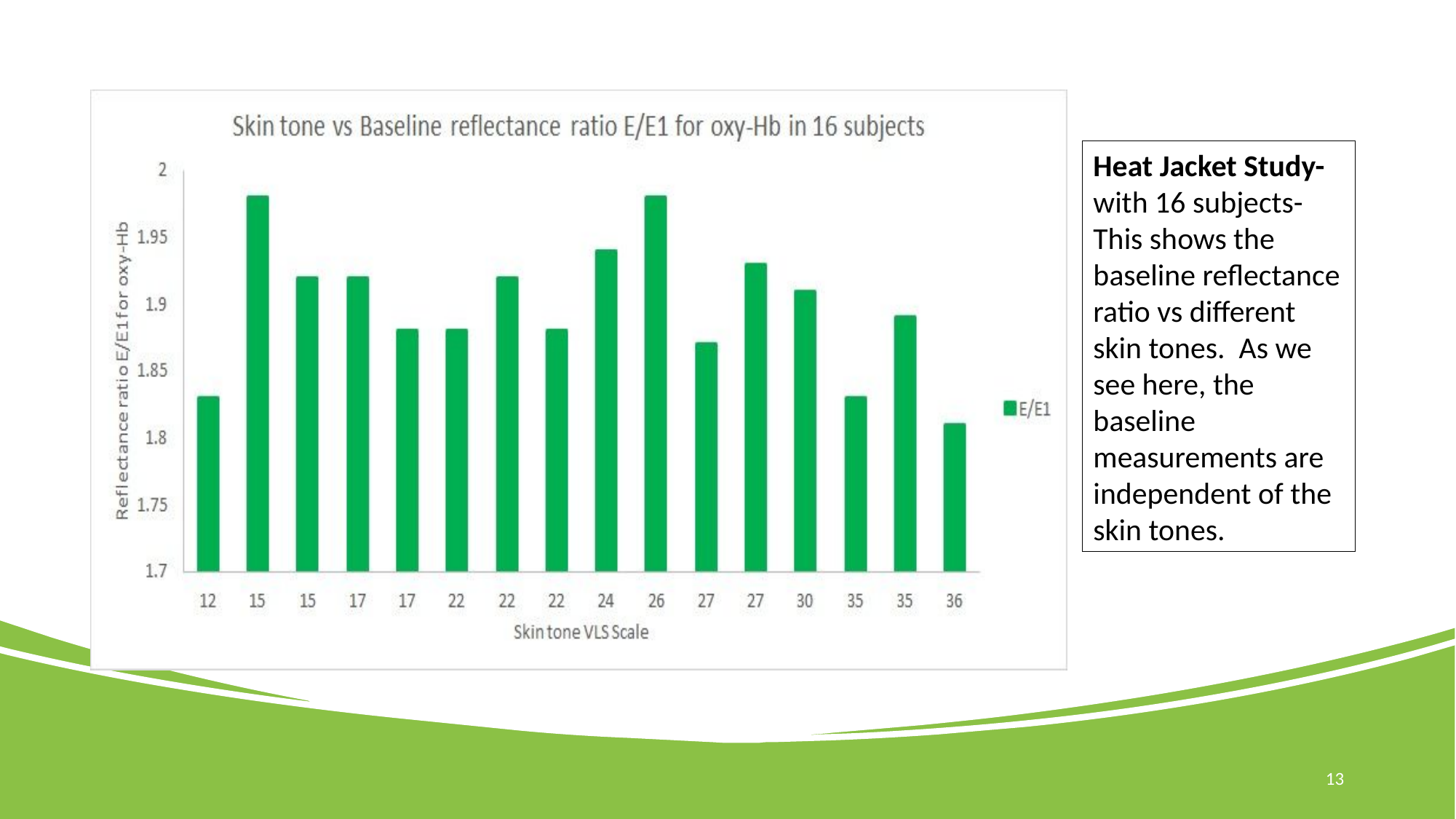

Heat Jacket Study-
with 16 subjects- This shows the baseline reflectance ratio vs different skin tones. As we see here, the baseline measurements are independent of the skin tones.
13

## Slide 14
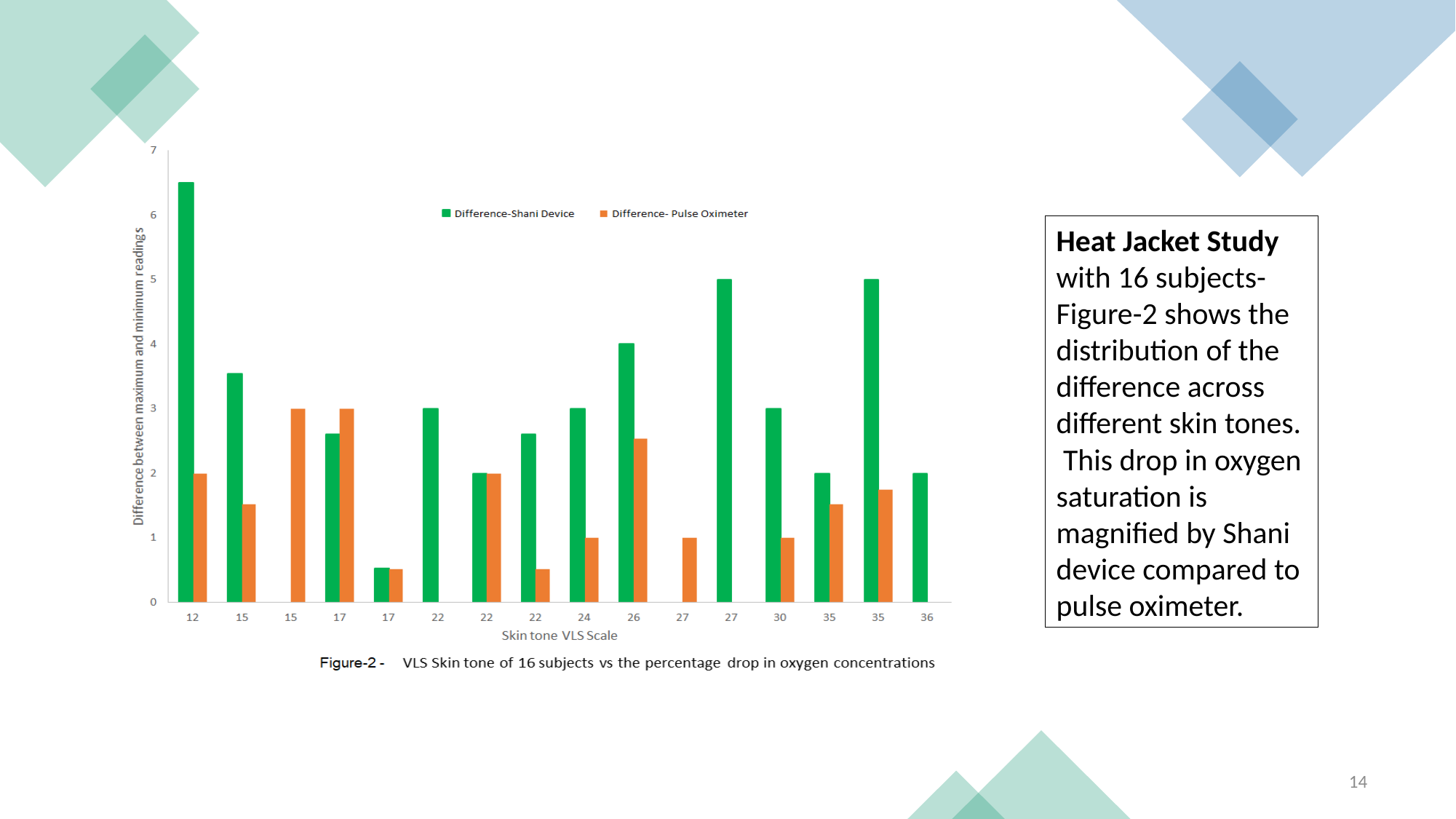

Heat Jacket Study with 16 subjects- Figure-2 shows the distribution of the difference across different skin tones. This drop in oxygen saturation is magnified by Shani device compared to pulse oximeter.
14

## Slide 15
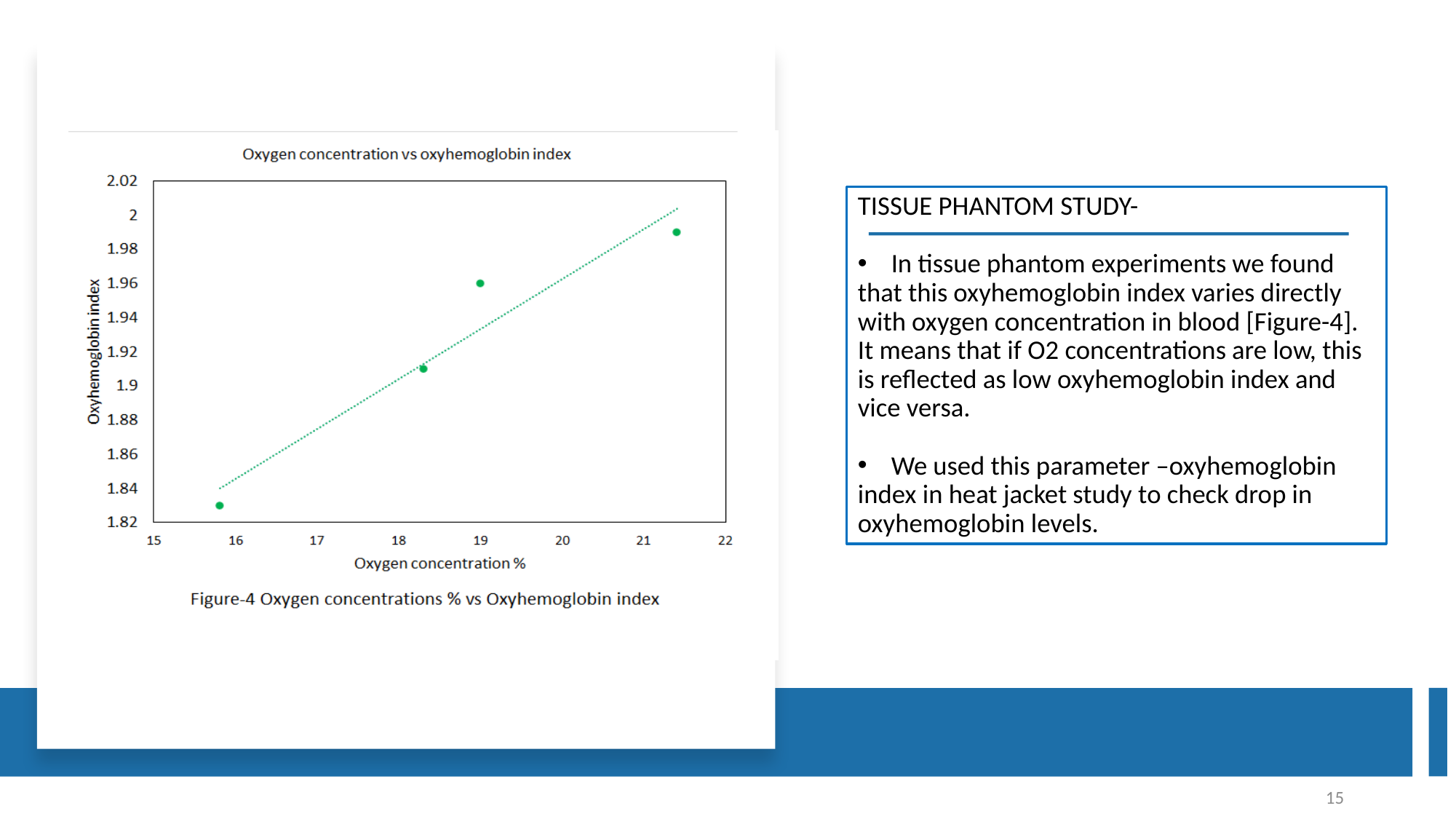

TISSUE PHANTOM STUDY-
In tissue phantom experiments we found
that this oxyhemoglobin index varies directly with oxygen concentration in blood [Figure-4]. It means that if O2 concentrations are low, this is reflected as low oxyhemoglobin index and vice versa.
We used this parameter –oxyhemoglobin
index in heat jacket study to check drop in oxyhemoglobin levels.
15

## Slide 16
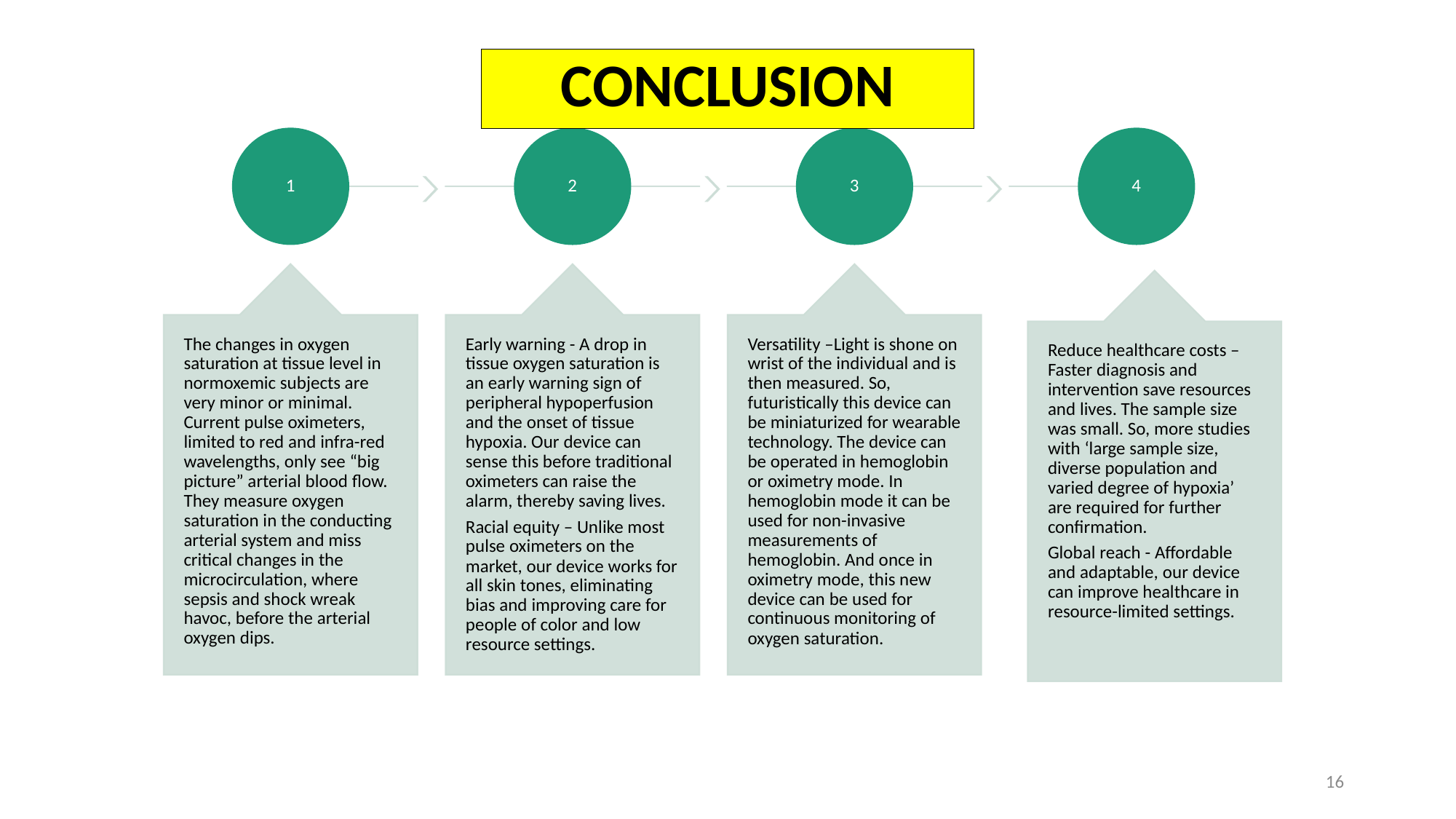

CONCLUSION
16
